# Development and Application of a Multiplex Reverse Transcription–Droplet Digital PCR Assay for Simultaneous Detection of Hepatitis A Virus and Hepatitis E Virus in Bivalve Shellfish

**DOI:** 10.3390/foods14010002

**Published:** 2024-12-24

**Authors:** Maolin Wei, Jinfeng Wang, Yan Wang, Libing Liu, Xiangdong Xu, Jianchang Wang

**Affiliations:** 1 School of Public Health, Hebei Medical University, Shijiazhuang 050017, China; wwml0927@163.com (M.W.); wy091468@163.com (Y.W.); 2 Food Microbiology and Animal Quarantine Laboratory, Technology Center of Shijiazhuang Customs, Shijiazhuang 050051, China; jinfengwang0625@163.com (J.W.); bing521564@163.com (L.L.); 3 Hebei Key Laboratory of Environment and Human Health, Shijiazhuang 050017, China

**Keywords:** MS2 phage, multiplex, reverse transcription–droplet digital PCR, hepatitis A virus, hepatitis E virus, bivalve shellfish

## Abstract

Foodborne viruses are significant contributors to global food safety incidents, posing a serious burden on human health and food safety. In this study, a multiplex reverse transcription–droplet digital PCR (RT-ddPCR) assay based on the MS2 phage as a process control virus (PCV) was developed to achieve the simultaneous detection of hepatitis A virus (HAV) and hepatitis E virus (HEV) in bivalve shellfish. By optimizing the reaction system and procedures, the best reaction conditions were selected, and the specificity, sensitivity, and reproducibility of the method were assessed. Additionally, the MS2 phage’s recovery rate was utilized as an indicator to evaluate the optimal sample nucleic acid enrichment method. The results indicated that the RT-ddPCR assay exhibited optimal amplification efficiency with primer concentrations of 900 nmol/L, probe concentrations of 350 nmol/L for HAV and HEV, and 500 nmol/L for MS2, an annealing temperature of 53.1 °C, an extension time of 90 s, and 45 cycles. Additionally, the developed multiplex RT-ddPCR assay demonstrated high specificity, with quantitation limits of 12.6, 8.9, and 7.8 copies/reaction being observed for HAV, HEV, and the MS2 phage, respectively. A total of 240 bivalve samples were analyzed, of which 4 were positive for HAV and 12 for HEV. The viral loads for HAV ranged from 3048 to 6528 copies/2 g, while those for HEV ranged from 3312 to 20,350 copies/2 g. This assay provides a vital tool for enhancing food safety monitoring.

## 1. Introduction

As living standards improve and consumers demand a greater variety of food options, the global demand for seafood products is increasing. This trend is attributed to their high nutritional value and unique flavor. However, seafood is susceptible to contamination by pathogens during the different stages of its production, processing, and handling, which renders it a significant vector for the transmission of foodborne pathogens [1,2]. It is estimated that 600 million cases of foodborne diseases occur annually, resulting in 420,000 deaths globally [3]. This represents a significant public health concern and a crucial factor in ensuring food safety. A systematic review covering 32 years (from 1980 to 2012) revealed 359 outbreaks of foodborne viruses linked to bivalve shellfish [4]. A hepatitis A outbreak took place from January to March 2020, confirmed through an epidemiological investigation and molecular sequencing to be closely related to the consumption of contaminated *Ostreidae* in Yantai City, Shandong Province, China [2]. Bivalve shellfish is a common seafood product with a high nutritional value and is recommended for consumption of high-quality proteins. However, contamination occurs because pathogens accumulate in bivalve organisms in the digestive glands from the water column during the feeding process. As a result, consumers who eat raw or undercooked bivalve products may be at risk of contracting viruses such as hepatitis A virus (HAV), hepatitis E virus (HEV), and norovirus (NoV) [5,6,7,8]. All of these foodborne viruses can be transmitted through the fecal–oral route and are regarded as important viral pathogens concerning foodborne diseases [9].

HAV and HEV are the primary viruses responsible for hepatitis infections in both developed and developing nations, with contaminated seafood and water serving as the main transmission sources to humans [10]. Additionally, HEV can also be transmitted zoonotically through the consumption of infected meat or meat products [11]. Both HAV and HEV are small RNA viruses, yet they exhibit notable differences in their genomes and structural characteristics [12]. HAV is a positive-stranded RNA virus with a diameter of 27–32 nm that causes acute liver disease. The disease presents with a range of symptoms, including fever, malaise, loss of appetite, diarrhea, nausea, and abdominal discomfort [13,14]. HEV is a non-enveloped virus with a single-stranded RNA genome measuring 7.2 kb in length. It contains three partially overlapping open reading frames (ORFs) with short 5′ and 3′ untranslated regions [15]. Most HEV infections are asymptomatic, yet HEV can cause symptomatic infections, resulting in self-limiting acute hepatitis [16]. HAV and HEV are distributed worldwide. Over the past 20 years, Europe has reported more than 21,000 acute clinical cases attributed to HEV, marking an overall 10-fold increase. According to the World Health Organization, around 7134 people died from HAV globally in 2016, representing 0.5% of the mortality rate from viral hepatitis. The infection rate among children is relatively high, and most remain asymptomatic; however, they can act as potential sources of infection as carriers of the virus. Hepatitis A and E exhibit higher incidence rates in regions with poor sanitation and relatively underdeveloped economies, displaying endemic characteristics [11,17,18]. In special populations, including pregnant women, the elderly, and individuals with pre-existing liver disease, infections frequently lead to more severe illness. Pregnant women infected with HAV or HEV, especially in the later stages of pregnancy, face a heightened risk of acute liver failure, which can result in adverse pregnancy outcomes such as premature birth and stillbirth, posing a significant threat to both maternal and infant health [19].

The current methods for detecting foodborne viruses include reverse transcription–polymerase chain reaction (RT-PCR) [20], reverse transcription–quantitative PCR (RT-qPCR) [21], reverse transcription–droplet digital PCR (RT-ddPCR) [22], and electrochemical immunosensor [23]. One of the most frequently utilized methodologies is RT-qPCR. However, the effectiveness of this assay is susceptible to the presence of inhibitory substances in the sample, the efficiency of reverse transcription, and the requirement for a standard curve [24]. The viral load of HAV and HEV in shellfish is low, which presents a significant challenge in meeting the demand for detection. Additionally, the substances present in the environmental media of shellfish, such as seawater and silt, are complex and may introduce a degree of interference in the detection process. It is therefore imperative that an accurate, efficient, and highly sensitive quantitative detection assay for shellfish samples be developed.

The ddPCR method, a third-generation PCR technique, permits the absolute quantification of nucleic acids in a sample without recourse to a standard curve. The method employs a droplet generator to randomly produce tens of thousands of droplets, which contain oil and the nucleic acids of the samples to be detected. Following amplification using a PCR machine, the number of droplets is assessed with a droplet reader, enabling the absolute quantification of the target gene [25,26]. Compared to qPCR, ddPCR shows greater resistance to PCR inhibitors, requires no standard curve for the absolute quantification of target nucleic acids, and demonstrates improved reproducibility at low concentration [27,28]. As a supplementary methodology to PCR, ddPCR has been extensively utilized in diverse domains, including the quantitative detection of transgenic elements [29], the absolute quantitative assessment of viral load [30], and gene expression analysis [31]. The MS2 phage was utilized in this study as a process control virus (PCV) to develop a highly efficient, specific, and sensitive triple RT-ddPCR assay for the simultaneous detection of HAV and HEV. The assay was effectively used for the quantitative detection of HAV and HEV in bivalves from various geographical regions within Hebei Province in China.

## 2. Materials and Methods

### 2.1. Viruses, Bacteria, and Samples

Nucleic acids for HAV, HEV, NoV genogroup I (GI) and genogroup II (GII), human astrovirus (HAstV), and sapovirus (SaV) were preserved at the Technology Center of Shijiazhuang Customs. The strains of *Vibrio parahaemolyticus* (CICC 21617), *Staphylococcus aureus* (CICC 10306), *Escherichia coli* (CICC 10899), and *Salmonella* (CICC 22956) were obtained from the China Industrial Microbial Strain Preservation Management Center (CICC). The *E. coli* strain (ATCC 15597) was sourced from the American Type Culture Collection (ATCC), while the MS2 phage (ATCC 15597-B1) was generously provided by the Technology Center of Qingdao Customs.

A total of 240 samples of commercially available bivalve shellfish were collected at farmers’ markets in various areas of Hebei Province (which has a long coastline and borders the Bohai Sea) from October 2023 to August 2024, including 84 *Ruditapes philippinarums*, 43 *Ostreidaes*, 63 *Pectinidaes*, 27 *Sinonovacula constrictas*, and 23 *Scapharca subcrenatas*.

### 2.2. MS2 Phage Culture and Potency Assay

In accordance with the methodology outlined in the literature [32], the MS2 phage was cultivated using the double-layer agar method. The MS2 phage suspension was diluted in a 10-fold gradient, and three plates were inoculated at each dilution. The plates were incubated for 24 h according to the aforementioned method. The potency of the MS2 phage was determined by counting the number of phage plaques falling within the range of 30 to 300. The potency was calculated using the following formula: potency [plaque forming unit (PFU)/mL] = average number of PFU ÷ volume of inoculation (mL) × dilution times.

### 2.3. Primers and Probes Used in the RT-ddPCR Assay

The sequences of the primers and probes for HAV, HEV, and MS2 used in the current study are shown in Table 1. The TaqMan probe for HAV was labeled with the VIC fluorescent group, while HEV was labeled with the FAM fluorescent group. The two probes for MS2 were labeled with VIC and FAM fluorescent groups, respectively. All the primers and probes were synthesized by Sangon Biotechnology (Shanghai, China).

### 2.4. Construction of HAV, HEV, and MS2 RNA Transcripts

The HAV target fragment (174 bp), HEV target fragment (160 bp), and MS2 target fragment (202 bp) sequences were artificially synthesized into pGEM-HAV, pGEM-HEV, and pGEM-MS2 plasmids, respectively, by Genecefe Biotech in Jiangsu, China. Then, the pGEM-HAV, pGEM-HEV, and pGEM-MS2 plasmids were linearized using NdeI (TaKaRa, Dalian, China) and subsequently transcribed into RNA with the T7 RiboMAX™ Express Large Scale RNA Production System (Promega, Madison, WI, USA). The in vitro transcription mixture was treated with RNase-Free DNaseI (Tiangen BioTech, Beijing, China) to eliminate any residues and then purified using the RNA clean kit (Tiangen BioTech, Beijing, China). The concentration of the RNA transcripts was measured using the NanoDrop 2000c (Thermo Scientific, Waltham, MA, USA).

### 2.5. RT-ddPCR Assay

The multiplex RT-ddPCR method was developed using Bio-Rad’s One-step RT-ddPCR advanced probe kit (Hercules, CA, USA) in a reaction volume of 20 μL. The specific reaction system is detailed in Table 2. The thermal cycling conditions were as follows: 50 °C for 60 min for the reverse transcription; 95 °C for 10 min for the enzyme activation; denaturation at 94 °C for 30 s; annealing temperature of 53.1 °C; and an extension time at 90 s for 45 cycles. This was followed by enzyme deactivation at 98 °C for 10 min and storage at 4 °C. The ramp rate utilized was 1 °C/s. The 96-well PCR plate was then transferred to the QX200™ Droplet Reader for analysis using QuantaSoft™ version 1.7.4 or QuantaSoft™ Analysis Pro version 1.0.596.

In order to optimize the condition, two key performance parameters were evaluated: the number of “raindrops” (i.e., droplets that form between clusters of negative and positive droplets) and the resolution (i.e., well-separated droplet clusters) [36]. To determine the best conditions, the assay was optimized in this study using the single variable approach, which looked at the primer final concentrations (500, 700, 900, and 1100 nmol/L), the probe final concentrations (150, 250, 350, and 500 nmol/L), the annealing temperature (51.0, 51.8, 53.1, 55.2, 57.7, 59.8, 61.2, and 62.0 °C), extension time (60 s, 90 s, and 120 s), and cycle number (40 and 45).

The single RT-ddPCR assay for HEV was carried out following the developed protocol detailed in the literature [34], while the single HAV RT-ddPCR assay was executed in accordance with the developed literature [37].

### 2.6. Analytical Specificity and Sensitivity Analysis

A multiplex RT-ddPCR assay specificity analysis was conducted using in vitro transcribed HAV, HEV, MS2 RNA, and RNA/DNA of common foodborne microorganisms, including NoV GI and GII, HAstV, SaV, *Vibrio parahaemolyticus*, *Staphylococcus aureus*, *E. coli*, and *Salmonella*. Three independent specificity analyses were conducted using the aforementioned templates.

The sensitivity of the RT-ddPCR assay was assessed by conducting a 10-fold serial dilution from 10^5^ to 10^−1^ copies/reaction of HAV, HEV, and MS2 RNA, along with a 5-fold dilution of 10^2^ copies/reaction, with three repetitions for each dilution. The limit of quantification (LOQ) represents the lowest target copy number in a sample that can be quantified reliably with acceptable precision and accuracy. The LOQ of each target in the RT-ddPCR system was estimated as the lowest copy number within the linear range with a relative standard deviation (RSD) of the measured copy number ≤25%. Thereafter, additional 2-, 4-, and 8-fold dilutions of 10^1^ copies/reaction were conducted, with 10 replicates per dilution series. The probit regression analysis was conducted on the data from each sample in each of the 10 replicates in order to ascertain the 95% limit of detection (LOD) [38]. In the analysis, nuclease-free water was employed as a negative control (NC).

### 2.7. Comparison of Viral RNA Enrichment Methods in Bivalve Shellfish

The digestive glands of bivalve shellfish (*Ruditapes philippinarums* and *Ostreidaes*) were subjected to each of the following three viral enrichment methods. Subsequently, the nucleic acid extraction was conducted on 280 μL of the resulting virus-enriched supernatant using the QIAamp Viral RNA Mini Kit (Qiagen, Germantown, MD, USA), following the manufacturer’s instructions. The extracted RNA was eluted with 60 μL of RNase-free water and stored at 80 °C or used immediately. The multiplex RT-ddPCR assay outlined in Section 2.5 was employed for the detection process, with each sample arranged in two parallel groups and repeated three times.

#### 2.7.1. Method 1: Proteinase K

Referencing and enhancing the ISO 15216-2:2019 [33] method, 2.0 g of bivalve digestive glands was placed into a 50 mL sterile centrifuge tube. The glands were homogenized using a homogenizer, and 10 μL of PCV (MS2 phage) was added to the homogenate. Following this, 1 mL of PBS and 10 μL 20 mg/mL of proteinase K solution (Tiangen BioTech, Beijing, China) were added, and the mixture was vortexed for 30 s. It was then shaken with a shaker at 37 °C at 320 rpm for 60 min, followed by a 15 min incubation in a water bath at 60 °C. Thereafter, the mixture was centrifuged at 12,000 rpm for 10 min at room temperature, and the supernatant (volume *V*_3_ was recorded) was collected into a centrifuge tube.

#### 2.7.2. Method 2: Proteinase K + Trizol/Chloroform

The supernatant obtained from Method 1 using proteinase K was combined with an equal volume of Trizol reagent (Ambion, Austin, TX, USA). Thorough mixing was ensured, vigorous agitation was applied, and the mixture was allowed to stand at room temperature for 5 min. Afterward, 0.3 times the volume of chloroform (Tianjin Yongda Chemical Reagent, Tianjin, China) was added, the mixture was vigorously vortexed for 30 s, and centrifugation was conducted at 12,000 rpm for 5 min at 4 °C. Finally, the upper aqueous phase was transferred to a new centrifuge tube, noting the volume *V*_3_.

#### 2.7.3. Method 3: Proteinase K + 5 × PEG8000 + Chloroform/n-Butanol

The nucleic acids were enriched as referenced [39]. To the supernatant obtained from the proteinase K method, 1/4 of the volume of 5 × PEG8000 solution was added, and thorough mixing was performed using a vortex mixer. Then, the solution was incubated for 60 min at 60 rpm and 4 °C, after which centrifugation was conducted at 10,000 rpm for 30 min at the same temperature. The supernatant was discarded, and the pellet was fully resuspended in 700 μL of PBS solution. An equal volume of a 1:1 chloroform–n-butanol solution (Tianjin Yongda Chemical Reagent, Tianjin, China) was added to the PBS suspension, and thorough shaking was performed before incubation at room temperature for 5 min. Later, centrifugation was conducted at 10,000 r/min for 15 min at 4 °C, and the upper aqueous phase was collected (volume *V*_3_ was recorded) in a 1.5 mL centrifuge tube.

### 2.8. Quantitative Detection of HAV and HEV in Bivalves

To validate the applicability of the developed multiplex RT-ddPCR assay for detecting real samples, the assay was employed for the quantitative detection of HAV and HEV in 240 shellfish samples. The procedure involved the addition of the MS2 phage as a PCV to monitor the entire detection process. The validity of the detection process and the actual virus content in the samples were assessed by calculating the recovery rate of MS2, and a qualitative comparison was made with the corresponding single RT-ddPCR assay. The formula for calculating the recovery rate is presented in Equation (1), while the formula for determining the viral content of HAV and HEV is shown in Equation (2).

(1) $$\mathrm{Recovery}\;\mathrm{rate}\;\mathrm{formula}:\;B=\frac{C_{MS2}\times A\times V_{1}}{C_{0}\times V_{2}}\times \frac{V_{3}}{280}\times 100\%$$

In Equation (1), *B* represents the recovery rate of the MS2 phage, expressed as a percentage; *C*_MS2_ denotes the concentration of MS2, calculated by multiplying the RT-ddPCR assay value of MS2 by the volume of the reaction system and dividing by the volume of RNA template in the reaction system, expressed in copies/μL; *A* indicates the dilution of the RNA template; *V*_1_ refers to the volume of RNA template after extraction, measured in μL; *C*_0_ represents the original concentration of MS2 added to the sample, expressed in copies/μL; *V*_2_ denotes the volume of MS2 added to the sample, measured in μL; and *V*_3_ indicates the total volume of supernatant after pre-treatment, expressed in μL.

(2) $$\mathrm{Viral}\;\mathrm{load}\;\mathrm{calculation}\;\mathrm{formula}:\;D=\frac{C_{Mean}\times A\times V_{1}}{B}$$

In Equation (2), *D* is defined as the viral load, expressed in copies/2 g; *C*_Mean_ denotes the average of sample test results, measured in copies/μL; *A* represents the dilution of the RNA template; *V*_1_ refers to the volume of the RNA template after extraction, measured in μL; and *B* indicates the recovery rate of the MS2 phage, expressed as a percentage.

### 2.9. Statistical Analysis

The statistical analysis was conducted using SPSS software (SPSS v21.0, IBM, Chicago, IL, USA). The comparison of two groups of data was performed using the *t*-test, while a one-way ANOVA was applied for comparisons involving three or more groups. Cohen’s Kappa statistic was employed to assess the agreement between the qualitative results of single RT-ddPCR and multiplex RT-ddPCR (*α* = 0.05).

## 3. Results

### 3.1. Optimization of Multiplex RT-ddPCR

**Figure 1:**
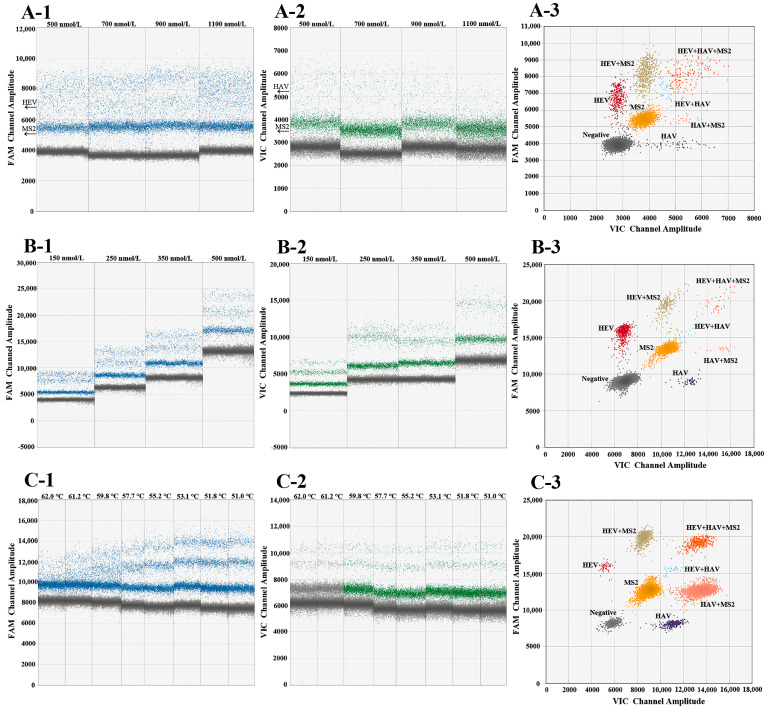
Optimization of the multiplex RT-ddPCR assay. (**A-1**,**A-2**) The FAM channel (MS2 and HEV) and the VIC channel (MS2 and HAV) primer concentrations’ optimization. (**A-3**) The 2D plot of the optimal primer final concentration for MS2, HEV, and HAV at 900 nmol/L. (**B-1**,**B-2**) The FAM channel (MS2 and HEV) and the VIC channel (MS2 and HAV) probe concentrations’ optimization. (**B-3**) The 2D plot when the optimal probe concentrations were 350 nmol/L for HAV and HEV and 500 nmol/L for MS2. (**C-1**,**C-2**) The FAM channel (MS2 and HEV) and the VIC channel (MS2 and HAV) annealing temperature optimization. (**C-3**) The final 2D plot after optimization of the multiplex RT-ddPCR assay.

**Figure 2:**
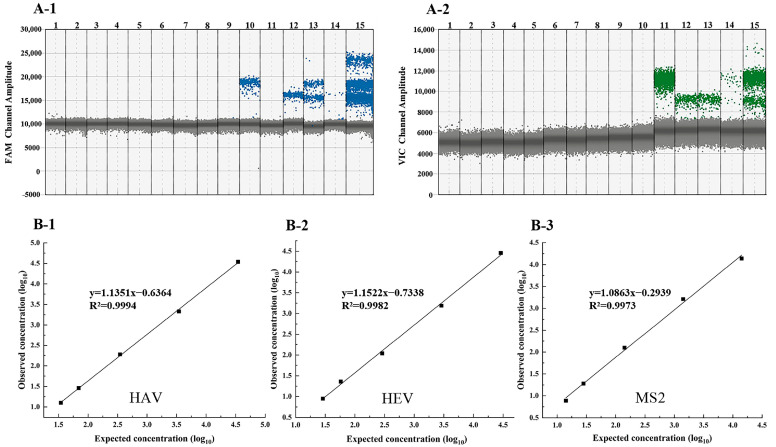
Specificity and linear range of multiplex RT-ddPCR assay. (**A-1**) The FAM channel specificity assay. (**A-2**) The VIC channel specificity assay. 1: ddH_2_O (−); 2: NoV GI (−); 3: NoV GII (−); 4: HAstV (−); 5: SV (−); 6: *Vibrio parahaemolyticus* (−); 7: *Staphylococcus aureus* (−); 8: *Escherichia coli* (−); 9: *Salmonella* (−); 10: HEV (+), HAV (−), MS2 (−); 11: HAV (+), HEV (−), MS2 (−); 12: MS2 (+), HEV (−), HAV (−); 13: HEV (+), MS2 (+), HAV (−); 14: HAV (+), MS2 (+), HEV (−); 15: MS2 (+), HEV (+), HAV (+). (+: detected; −: not detected). (**B-1**,**B-2**,**B-3**) The linear range of multiplex RT-ddPCR for the quantitative detection of HAV, HEV, and MS2 RNA.

**Figure 3:**
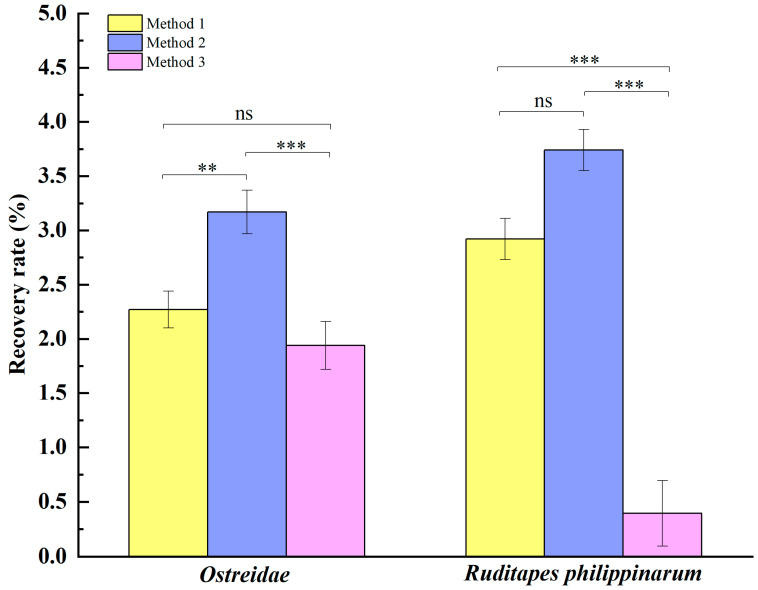
Comparison of the results of 3 different treatments (*n* = 6). Note: **: *p* < 0.01; ***: *p* < 0.001; ns: *p* > 0.05.

Due to the numerous target gene sequences in the multiplex RT-ddPCR assay, the amplification and separation efficiency were significantly affected by the final concentrations of the primer, probe, annealing temperature, extension time, and the number of cycles. Therefore, the reaction system and procedure were optimized. The final primer concentrations were based on their impact on amplification efficiency, with findings indicating that the optimal concentration was 900 nmol/L (Figure 1A). The probe final concentrations were optimized between 150 and 500 nmol/L, showing that the separation of negative and positive droplets was more distinct at 350 nmol/L for HAV and HEV. However, the spacing between MS2 positive and negative droplets was closer. Therefore, enhancing the MS2 probe concentration to 500 nmol/L significantly widened the spacing between negative and positive droplets (Figure 1B). Thus, the optimal probe concentrations for HAV and HEV were 350 nmol/L, while the optimal concentration for MS2 was 500 nmol/L. To determine the best annealing temperature, eight temperatures ranging from 51.0 to 62.0 °C were evaluated. The highest number of positive droplets was achieved at 53.1 °C, resulting in the most effective isolation (Figure 1C). Finally, by maintaining the number of cycles at 40 and optimizing the extension time (60, 90, 120 s), it was observed that the positive droplets were more compact and better separated with an extension time of 90 s and 45 cycles. The 2D plot analysis results of the optimized multiplex RT-ddPCR assay are illustrated in Figure 1C-3.

### 3.2. Analysis of Specificity and Sensitivity

Using various primer and probe combinations, the assay was used to detect one or more target genes and non-target genes to assess its specificity. The findings (Figure 2A) demonstrated that one or more target genes exhibited positive droplets, whereas no positive droplets were detected for any of the non-target genes. This indicated that the assay possessed a high level of specificity.

Following the mixing of three distinct types of RNA (HAV, HEV, and MS2), a ten-fold serial dilution was performed, with each dilution repeated three times. The results indicated that the linear ranges for HAV, HEV, and MS2 were 35 to 35,000, 29 to 29,000, and 14 to 14,000 copies/reaction, respectively. The LOQ were found to be 12.6, 8.9, and 7.8 copies/reaction, respectively. The coefficients of determination (R^2^) for the target concentrations of HAV, HEV, and MS2 were recorded as 0.9994, 0.9982, and 0.9973, respectively (Figure 2B). The reproducibility of the quantitative linear ranges was found to be between 3.2% and 18.8%, which is below the acceptable threshold for quantitative methods (<25%) [33].

The LOD was defined as the lowest template concentration that could be detected with a 95% probability of positivity, and the assay was based on the results of 10 replicates. We determined the 95% LOD using a probit regression analysis, which can convert a linear combination of independent variables into probability values through the cumulative distribution function of a normal distribution. This method effectively addresses the issue of probability estimation, making the results more meaningful in practice. The results for HAV, HEV, and MS2 are presented in Table 3. The concentration of the assay at the 95% probability level was determined through the probit regression analysis, resulting in an LOD of 9.66 copies/reaction (95% CI: 7.65 to 17.16 copies/reaction) for HAV and 8.42 copies/reaction (95% CI: 7.11 to 11.93 copies/reaction) for HEV. The LOD for MS2 was found to be 6.91 copies/reaction (95% CI: 5.38 to 11.30 copies/reaction).

### 3.3. Viral Nucleic Acid Enrichment Method

Preparation of artificially contaminated samples using the MS2 phage was carried out successfully. The *E. coli* MS2 phage was prepared using the double-layer agar culture method, and the potency of this MS2 phage suspension was determined to be 8.1 × 10^11^ PFU/mL after a dilution of 10^9^. This suspension was then serially diluted 10-fold and set aside.

To prepare the samples, 10 μL of MS2 phage (2.0 × 10^4^ copies/μL) was added to 2.0 g of digestive glands from *Ruditapes philippinarums* and *Ostreidaes*. These samples were then subjected to three different enrichment methods. After treatment, nucleic acids were extracted using a viral RNA extraction kit, and each method was performed twice in parallel. Finally, the multiplex RT-ddPCR assay was utilized for three repetitions for detection, and a statistical analysis was conducted on the recovery rate of the MS2 phage.

The recovery rates for the MS2 phage after treatment with different enrichment methods were calculated based on Formula 2.8 (1). The homogeneity of variance was confirmed (*p* > 0.05), and the one-way ANOVA test–Duncan method was chosen for two-by-two comparisons. As shown in Figure 3, the MS2 phage recoveries obtained from *Ostreidaes* after treatment with the three enrichment methods were compared by two-by-two comparison. It was found that the *p <* 0.01 between Method 2 and Methods 1 and 3 indicated statistically significant differences, with Method 2 being superior to Methods 1 and 3. In *Ruditapes philippinarums*, however, the *p <* 0.05 between Methods 2 and 1, along with the *p <* 0.001 between Methods 2 and 3, indicated that Methods 1 and 2 were superior to Method 3. Overall, the proteinase K + Trizol/chloroform method of Method 2 demonstrated higher recovery while saving time and effort, so the proteinase K + Trizol/chloroform method was chosen as the pre-treatment enrichment method for shellfish samples.

### 3.4. Detection of HAV and HEV in Shellfish

In this study, 240 shellfish samples were examined. The results indicated that multiplex RT-ddPCR identified 4 positive samples for HAV (1.67%) and 12 positive samples for HEV (5.00%), while single RT-ddPCR detected 4 positive samples for HAV (1.67%) and 15 positive samples for HEV (6.25%). A high level of agreement between multiplex and single RT-ddPCR assays was observed (HAV: kappa = 1.000, HEV: kappa = 0.882), with significant consistency (*p* < 0.001), suggesting that the developed multiplex RT-ddPCR assay had similar performance to the single RT-ddPCR assay for detecting HAV and HEV in bivalve samples. Out of the four HAV-positive samples detected by the multiplex RT-ddPCR assay, two were quantifiable, with concentrations ranging from 3048 to 6528 copies/2 g. Among the 12 HEV-positive samples, 4 were quantifiable, with concentrations ranging from 3312 to 20,350 copies/2 g. The recovery rate of the MS2 phage during the detection process was greater than 1% [33], indicating that the detection results were acceptable.

Among the 240 shellfish samples, there were 84 *Ruditapes philippinarums*, 43 *Ostreidaes*, 63 *Pectinidaes*, 27 *Sinonovacula constrictas*, and 23 *Scapharca subcrenatas*. The detection rates of HAV and HEV in *Ruditapes philippinarums* were reported as 1.19% (1/84) and 3.57% (3/84), respectively. In *Ostreidaes*, the detection rates were 6.98% (3/43) for HAV and 9.30% (4/43) for HEV. In *Pectinidaes*, no detection was reported for HAV, while a detection rate of 3.17% (2/63) for HEV was noted. The detection rates of HAV and HEV in *Sinonovacula constrictas* were non-detected and 3.70% (1/27), respectively, while in *Scapharca subcrenatas*, no detection was reported for HAV and a detection rate of 8.70% (2/23) for HEV was observed.

## 4. Discussion

A significant burden on human health and food safety has been posed by foodborne viruses, which have emerged as crucial pathogens responsible for global food safety incidents. Many cases of diseases have been linked to the consumption of shellfish contaminated with HAV or HEV [40,41]. However, the low viral load characteristics of HAV and HEV often lead to underestimated detection rates. Therefore, developing a method with high sensitivity and robust quantification capability is essential. Compared to qPCR, ddPCR is recognized for offering superior accuracy in low-copy samples and better tolerance to PCR inhibitors. Additionally, the multiplex ddPCR assay can reduce costs, time, and sample template requirements compared to single ddPCR.

In this study, a triple RT-ddPCR assay was developed and evaluated. This assay was based on the probe ratio approach, where the third target (MS2) was simultaneously labeled with two fluorescent groups, FAM and VIC, allowing the droplets to emit fluorescence signals at different wavelengths. Relative concentrations of 100% for FAM and VIC labeled probes were assigned to HEV and HAV, respectively, while a relative concentration of 50% for both FAM and VIC labeled probes was assigned to MS2. The predicted positions of the MS2 single positive clusters were typically found between the two-dimensional axes. When the three positive clusters were included, there were a total of eight different cluster combinations for the three targets [42]. The ISO 15216-2:2019 standard indicates that when PCR technology is employed for the detection of foodborne viruses, it is imperative that culturable non-enveloped positive-sense single-stranded RNA viruses are utilized for the purpose of process quality control. The MS2 phage is a non-enveloped positive-sense single-stranded RNA virus that is capable of infecting F+ (male) *E. coli*. The properties and structure of the MS2 phage are similar to those of the viruses being tested, but its genetic sequence differs significantly from that of the target viruses [43,44]. Consequently, it is able to simulate the handling and detection processes of authentic viral samples, thereby facilitating the assessment of the accuracy and reliability of laboratory detection methods and workflows. The MS2 phage was employed as a process control for the detection of HAV in water and food samples and was also used in monitoring NoV in fecal samples [45,46]. The results of both studies indicated that the MS2 phage provided a highly reliable and straightforward method for monitoring false-negative results, thus establishing it as a valuable tool in routine diagnostic laboratories. In this study, the MS2 phage was selected as the process control virus due to the absence of overlap between its genomic sequence and that of standard food samples, coupled with its resistance to digestion by both Dnase and Rnase. This renders it an effective monitor of the virus enrichment and RNA extraction processes during RNA virus detection and PCR amplification, allowing for the tracking of false negatives. The evaluation of the recovery rate of the MS2 phage demonstrated rates exceeding 1%, indicating that the sample processing, extraction, and purification steps during the experiment were relatively successful, allowing for the effective recovery of the MS2 phage. A recovery rate greater than 1% also reflected the proficiency of the laboratory operations and the optimization of experimental conditions, thereby fulfilling the experimental requirements.

Research indicates [47,48] that intermediate fluorescence values are generated by the ddPCR method between two clusters of droplets, which manifest as a “rain” pattern in the resulting graphs. This frequently complicates the differentiation between positive and negative droplets, resulting in erroneous outcomes. Therefore, the reduction in this “rain” is considered crucial. In this study, the reaction system and conditions of a multiplex RT-ddPCR assay were optimized by redesigning traditional TaqMan probes in the standards as dual-quenched TaqMan probes with two quenching groups, one at the 3′ end and the other centrally positioned within the probe. When two quenched probes are situated in a particular area, binding with one another is enabled, thereby achieving quenching and signal production. The design of the dual-quenched probes necessitates that both probes bind simultaneously in order to generate a signal, thereby reducing the probability of non-specific binding and background signal generation. This enhances the specificity and sensitivity of the PCR reaction [49,50]. It was revealed in the study that the use of traditional TaqMan probes resulted in a high background fluorescence signal intensity, which narrowed the gap between positive and negative droplets and impacted result interpretation. Conversely, the design of the probes as dual-quenched TaqMan probes decreased the background signal, enabling clear differentiation between positive and negative droplets. Following the optimization of the reaction system and conditions of the multiplex RT-ddPCR assay, an enhancement in resolution was observed, accompanied by a reduction in the formation of “rain”, resulting in high specificity and sensitivity. The LOQ for HAV, HEV, and MS2 was determined to be 12.6, 8.9, and 7.8 copies/reaction, respectively, with a LOD of 9.66, 8.42, and 6.91 copies/reaction. A triple ddPCR method was developed by Han et al. [36] with a detection limit of 5 copies/reaction for HAV, while La Bella et al. [51] reported a detection limit of 6.8 copies/reaction for HEV using their dual RT-ddPCR method. The sensitivity of the detection methods employed in these studies was found to be comparable to that observed in the present research. Gao et al. [52] developed a quantitative real-time reverse transcription–recombinase polymerase amplification assay (RT-qRPA), achieving a LOD of 17.7 copies/reaction for HEV. Filipa-Silva et al. [53] established an RT-qPCR assay with a LOD of 10^2^ copies/reaction for HAV. In contrast, the multiplex RT-ddPCR assay established in this study demonstrated greater sensitivity and was capable of detecting viral nucleic acids at lower concentrations. These comparative results clearly indicate that the multiplex RT-ddPCR assay developed in this study offers significant advantages and can serve as an effective diagnostic tool for evaluating samples with low concentrations of HAV and HEV RNA, providing strong support for relevant detection efforts.

Due to the low viral load in shellfish and the presence of substances such as polysaccharides, fats, and protease inhibitors, these factors can impact enzyme activity during RNA extraction, potentially causing RNA degradation or damage. Therefore, the optimization of sample pretreatment enrichment methods is considered a crucial step before conducting molecular biology tests. In this study, various pretreatment enrichment methods were compared, and the results showed that the proteinase K + Trizol/chloroform method was superior to the proteinase K method and the proteinase K + 5 × PEG8000 + chloroform/n-butanol method. Although the proteinase K method was time-efficient (2 h), it could not eliminate the significant amounts of fat components present in bivalves [54]. The proteinase K + 5× PEG8000 + chloroform/n-butanol method took the longest (4.5 h), and when chloroform/n-butanol was introduced, the emulsion membrane was susceptible to breaking during centrifugation to collect the upper aqueous phase. The proteinase K + Trizol/chloroform method further lysed and removed lipids from the supernatant extracted by proteinase K using Trizol/chloroform, thereby enhancing the purity of RNA and the efficiency of virus extraction [55]. Due to the relatively short duration of the proteinase K + Trizol/chloroform method (2.5 h), along with its stability and higher recovery rate, this method was selected as the optimal enrichment technique for the pretreatment of bivalve samples in this study.

The multiplex RT-ddPCR assay yielded a detection rate of 1.67% (4/240) for HAV and 5.00% (12/240) for HEV among the 240 samples. A detection rate of 1.67% (4/240) for HAV and 6.25% (15/240) for HEV was achieved using the single RT-ddPCR assay. High concordance was demonstrated in the results of both assays for HAV and HEV in bivalves, with kappa values of 1.000 and 0.882, respectively. The detection of HEV was higher in the single RT-ddPCR assay than in the multiplex RT-ddPCR assay, which may be attributed to the more straightforward design of primers and probes in the single assay, which facilitates the achievement of high sensitivity. In the multiplex RT-ddPCR assay, the design of multiple sets of primers and probes within the same reaction system is required, as well as the optimization of reaction conditions to enable the detection of multiple target sequences concurrently. This may potentially result in a reduction in sensitivity. Nevertheless, the multiplex RT-ddPCR assay enables the concurrent quantification of multiple viruses, thereby reducing the time and effort required.

In this study, HAV RNA was identified in 240 samples, with a maximum viral load of 6528 copies/2 g. Likewise, HEV RNA was detected at levels of up to 20,350 copies/2 g, which was corroborated by previous research [56,57] that also documented contamination of shellfish with HAV and HEV. Consumption of raw or undercooked shellfish has been identified as a potential source of hepatitis A or E virus infection. Subsequently, a study is planned to further augment the sample size and enhance sample diversity by incorporating a wider range of matrices such as fruits, vegetables, and wastewater. A more profound and detailed analysis will be carried out regarding the sources, types, and collection times of the positive samples. That approach will significantly improve our comprehension of the virus’s transmission modalities across diverse environmental settings and conditions. Additionally, the monitoring of samples procured from different seasons and regions holds the potential to disclose latent epidemic tendencies, thereby facilitating a more accurate assessment of the potential risks that foodborne viruses pose to public health.

## 5. Conclusions

A highly precise, reliable, and sensitive multiplex RT-ddPCR assay using the MS2 phage as a PCV to detect HAV and HEV simultaneously was developed in this study. With this method, low viral loads of HAV and HEV were successfully detected, enabling early and dependable identification of viruses. The results from this multiplex RT-ddPCR assay demonstrated good consistency with that of the single RT-ddPCR assay, and there was no cross-reactivity with common foodborne pathogens, enhancing detection efficiency while lowering costs. The optimized viral nucleic acid enrichment strategy in this study not only reduced the detection time but also improved the recovery rate of viral nucleic acids, making it a promising and effective tool for detecting foodborne viruses. Given the significant advantages this method offers in the field of foodborne virus detection, our research team is actively expanding its application to other food testing areas. We hope that this method will facilitate the early screening of potential viral contamination, preventing contaminated food from entering market circulation, thereby reducing the risk of public health incidents caused by the transmission of foodborne viruses and providing robust technical support for regulatory agencies like customs to implement precise control and timely interruption of the circulation of problematic food.

## Figures and Tables

**Table 1:** Primer and probe sequences.

Name	Sequence, 5′→3′	Amplicon Size/bp	GenBank	Sources
HAV-F	TCACCGCCGTTTGCCTAG	174	M14707	[33]
HAV-R	GGAGAGCCCTGGAAGAAAG			
HAV-P	VIC-CCTGAACCTGCAGGAATTAA-MGB			
HEV-F	ACHCTRTTTAAYCTTGCTGAYAC	160	AY594199	[34]
HEV-R	CCTTRTCCTGCTGAGCRTTCTC			
HEV-P	FAM-CCGACAGAATTGATTTCGTCGGC-BHQ1			
MS2-F	GGCTGCTCGCGGATACCC	202	JF719743.1	[35]
MS2-R	TGAGGGAATGTGGGAACCG			
MS2-P1	FAM-ACCTCGGGTTTCCGTCTTGCTCGT-DUQ-TQ ^a^			
MS2-P2	VIC-ACCTCGGGTTTCCGTCTTGCTCGT-DUQ-TQ			

H: A or C or T; Y: C or T; R: A or G. ^a^ DUQ-TQ dual quenching probes.

**Table 2:** Multiplex RT-ddPCR reaction system.

20 μL RT-ddPCR Reaction	Volume/μL
Supermix (one-step RT-ddPCR)	5
Reverse transcriptase	2
300 mM DTT	1
HAV/HEV/MS2 forward primers (900 nmol/L)	0.6/0.6/0.6
HAV/HEV/MS2 reverse Primers (900 nmol/L)	0.6/0.6/0.6
HAV/HEV probes (350 nmol/L)	0.33/0.33
MS2 probes (FAM/VIC) (250/250 nmol/L) ^b^	0.17/0.17
RNase-free water	4.4
RNA	3

^b^ MS2 probe is marked with 50% FAM + 50% VIC.

**Table 3:** Repeated detection results of HAV, HEV, and MS2 in the multiplex RT-ddPCR assay (*n* = 10).

HAV		HEV		MS2	
Concentration (Copies/Reaction)	Positive Counts	Concentration (Copies/Reaction)	Positive Counts	Concentration (Copies/Reaction)	Positive Counts
350	10	290	10	140	10
35	10	29	10	14	10
17.5	10	14.5	10	7	9
8.75	9	7.25	8	3.5	7
4.38	3	3.63	1	1.75	1
3.5	2	2.90	0	1.4	1

## Data Availability

The original contributions presented in the study are included in the article, further inquiries can be directed to the corresponding authors.
